# Computationally efficient real-time interpolation algorithm for non-uniform sampled biosignals

**DOI:** 10.1049/htl.2015.0031

**Published:** 2016-05-11

**Authors:** Onur Guven, Amir Eftekhar, Wilko Kindt, Timothy G. Constandinou

**Affiliations:** 1 Department of Electrical and Electronic Engineering, Imperial College, South Kensington Campus, London SW7 2AZ, UK; 2 Texas Instruments Corporation, Delft, Netherlands

**Keywords:** interpolation, electrocardiography, signal sampling, medical signal processing, data acquisition, signal denoising, computationally efficient real-time interpolation algorithm, nonuniform sampled biosignals, electrocardiogram baseline drift removal, isoelectric baseline points, algorithm segments, piecewise linear equations, linear curvature, real baseline wander data acquisition, MIT-BIH Noise Stress Database, heartbeat, standard deviation, frequency 0.05 Hz to 0.7 Hz

## Abstract

This Letter presents a novel, computationally efficient interpolation method that has been optimised for use in electrocardiogram baseline drift removal. In the authors’ previous Letter three isoelectric baseline points per heartbeat are detected, and here utilised as interpolation points. As an extension from linear interpolation, their algorithm segments the interpolation interval and utilises different piecewise linear equations. Thus, the algorithm produces a linear curvature that is computationally efficient while interpolating non-uniform samples. The proposed algorithm is tested using sinusoids with different fundamental frequencies from 0.05 to 0.7 Hz and also validated with real baseline wander data acquired from the Massachusetts Institute of Technology University and Boston's Beth Israel Hospital (MIT-BIH) Noise Stress Database. The synthetic data results show an root mean square (RMS) error of 0.9 μV (mean), 0.63 μV (median) and 0.6 μV (standard deviation) per heartbeat on a 1 mV_p–p_ 0.1 Hz sinusoid. On real data, they obtain an RMS error of 10.9 μV (mean), 8.5 μV (median) and 9.0 μV (standard deviation) per heartbeat. Cubic spline interpolation and linear interpolation on the other hand shows 10.7 μV, 11.6 μV (mean), 7.8 μV, 8.9 μV (median) and 9.8 μV, 9.3 μV (standard deviation) per heartbeat.

## 1 Introduction

Interpolation is a method of constructing new data points within the range of a discrete dataset. It is a problem that dates back to ancient civilisations, which were known to use interpolation methods for analysing astronomical data [1]. The mathematical basis of this method was not defined till later, as in the work of Waring [2], which is today attributed to Lagrange.

Lagrange polynomials define the least degree of polynomial curves that pass through a given set of coordinates *x*_*i*_, *y*_*i*_. However, as the order of Lagrange polynomials increase, any small perturbations in coordinates results in large overshoots at the end points as known in the literature as the Runge phenomenon [3]. These oscillations may have no relation to the true nature of the overall function itself and without rigorous error monitoring higher-order polynomial interpolations degrade accuracy as well as increase complexity of the algorithm.

Later, cubic spline (third order) functions were defined [4]. These polynomials are smoothly connected to each other at the coordinates *x*_*i*_, *y*_*i*_ and since their continuous first and second derivatives exist everywhere, the overall generated curve is smooth. However, spline interpolation algorithms rely on matrix inversion techniques for computing coefficients. Therefore, they are computationally demanding, and though efficient, they are not adaptable to real-time systems without windowing techniques. More adaptable are Rifman's [5] and Keys’ [6] cubic convolution interpolation methods which involve fitting piecewise cubic polynomials (kernels) within intervals. Similar to cubic splines, these methods are computationally complex and not suitable for certain real-time system designs.

There are of course several algorithms and methods throughout the literature, aiming to approximate smoother curves and better fits. However, for real-time systems challenges still exist to balance complexity versus accuracy, and to allow adaptability to changing signal dynamics. The latter especially the case in biological signal applications.

One example of such a biological signal is the electrocardiogram (ECG). Being prone to interference from physiological and environmental sources has made ambulatory ECG, with a clinical accuracy, a challenge. Techniques exist to remove each of these noise sources; however, on occasions where signal integrity is crucial these methods do not meet clinical standards. As discussed in our previous work [7], baseline wander can be removed by detecting fiducial points and estimating the baseline wander by interpolating through those points with a piecewise cubic hermite interpolation (PCHIP). However, PCHIP is still somewhat complex; therefore, a new method is investigated where baseline wander estimation is accurately achieved with less computational hardware resources required.

This Letter presents a new interpolation algorithm that allows a better tradeoff between computational efficiency and signal distortion than prior methods. ECG signals are used as our test application, wherein we measure the distortion of the ST segment (an indicator of heart malfunction) while estimating the baseline wander. These baseline wander signals are low-frequency noise artefacts that can be modelled as sinusoids with amplitudes up to 300 μV. This Letter is organised as follows: Section 2 describes the overall system concept and methods; Section 3 describes the artificial and real test datasets; Section 4 presents and discusses results with complex algorithms; and Section 5 concludes this Letter.

## 2 Methodology

**Fig. 1:**
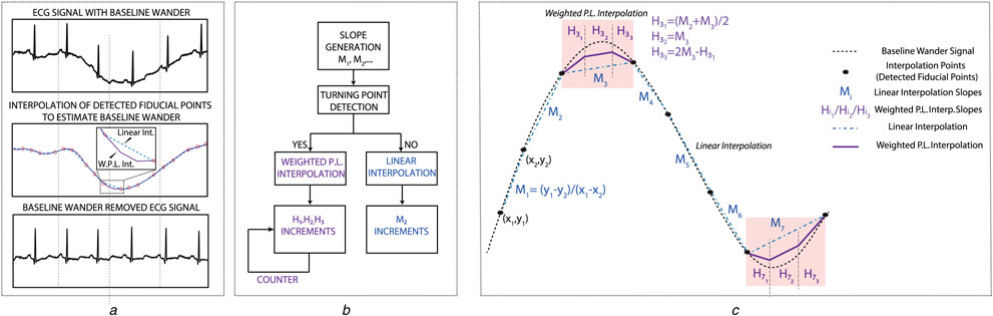
Weighted piecewise interpolation methodology showing *a* Example input signal *b* Algorithm flowchart *c* Illustration of concept

The overall methodology to the proposed algorithm is illustrated in Fig. 1. The main purpose of the algorithm is to estimate curvatures (turning points) with a better approximation than linear interpolation and in other cases simply use linear interpolation to reduce computational complexity. The algorithm is divided into two stages: (i) turning point detection and (ii) weighted piecewise linear (WPL) interpolation.

### 2.1 Turning point detection

First, we define the slopes, *M*_*i*_, between adjacent interpolation points. These slopes are then used to determine if a turning point exists. Here, we utilise two criteria. The first condition checks if the slopes change sign such that either a local/absolute minima or maxima exists. However, this condition on its own is not enough to capture all turning points such that on occasions, when adjacent slopes do not change sign, there might be possible curvatures such as during *M*_3_ instant as shown in Fig. 1. Therefore, a second condition is required such that even when the slopes do not change sign, these turning points are detected accurately. We have found that when the magnitude of adjacent slopes satisfies Condition 2 in (1), the algorithm accuracy improves even though no local/absolute minima or maxima are detected.

During noisy conditions, it is harder to detect interpolation points as for every other method. Therefore, filters have been utilised to crudely remove noise artefacts and detect these points as reported in our previous work [7]. Once these points are located, turning point conditions focus on reducing random errors associated with the interpolation method. On the contrary, when there is no turning point detection, the algorithm uses linear interpolation to improve computational efficiency of the overall algorithm

(1) $$\matrix{ {{\rm Condition}\; 1 \to {M_{i - 1}} \gt 0\; {\&}\; {M_i} \lt 0\ \vert \vert\ {M_{i - 1}} \lt 0\; {\&}\; {M_i} \gt 0} \hfill \cr {{\rm Condition}\; 2 \to \displaystyle{3 \over 4}\; \ast\; \vert {M_{i - 1}}\vert \gt \vert {M_i}\vert\ \vert\vert \displaystyle{3 \over 4}\;\ast\; \vert {M_i}\vert \gt \vert {M_{i - 1}}\vert } \hfill \cr } \eqno\lpar 1\rpar $$

### 2.2 Interpolation methods

#### 2.2.1 Linear interpolation

This method only requires current slope, *M*_*i*_, and a fraction of this slope is added for every interpolation point in between *y*_*i*_ and *y*_*i*+1_. Therefore, the number of operations required is minimal and the algorithm can interpolate both uniformly and non-uniformly sampled data since interpolation is based on addition operation and the only condition is to meet *x*_*i*+1_, *y*_*i*+1_ coordinates. In Fig. 1, linear interpolation occurs at intervals *M*_1,2,4,5,6_.

#### 2.2.2 WPL interpolation

An improvement to linear interpolation is achieved when a turning point is detected as shown in Fig. 1. Following this detection, the distance between *x*_*i*_ and *x*_*i*+1_ is calculated and this interval is divided into three equal smaller segments. A counter checks this segment distribution and on events where the distance cannot be divided accurately, a compensation factor is added to the final sample such that *x*_*i*+1_, *y*_*i*+1_ coordinates are met. As mentioned in linear interpolation, this characteristic shows that both uniformly and non-uniformly sampled data can be interpolated and in each of these segments WPL interpolation is achieved where every clock cycle, the corresponding segment slopes ${H_{{i_1}}}$, ${H_{{i_2}}}$, ${H_{{i_3}}}$ are added to the previous sample as such in linear interpolation. The first segment's slope, ${H_{{i_1}}}$, is the average of *M*_*i*−1_ and *M*_*i*_, which estimates the concavity/convexity with its past knowledge. The second slope, ${H_{{i_2}}}$ is defined as *M*_*i*_ and the last ${H_{{i_3}}}$ is shown as defined in (2). The error function of the WPL interpolation in this case are bounded by *M*_*i*_ and *M*_*i*+1_ slopes and though limited to three segments, the algorithm could be segmented further with increased complexity

(2) $${H_{{i_1}}} = \displaystyle{{{M_{i - 1}} + {M_i}} \over 2}\semicolon \; \; \; {H_{{i_2}}} = {M_i}\semicolon \; \; \; {H_{{i_3}}} = 2\ast {M_i} - {H_{{i_1}}}\eqno\lpar 2\rpar $$

## 3 Test data

To test our algorithm we use two sets of data: synthetic and real data. The former models ECG baseline wander, whereas the latter is real data that we shall describe. We first generate interpolation points that are realistic isoelectric fiducial points that define the baseline wander [7]. These fiducial points are generated over 2243 heartbeats of the Massachusetts Institute of Technology University and Boston's Beth Israel Hospital (MIT-BIH) Arrhythmia Database signal 100 m.mat. These points are therefore realistic representations of non-uniformly distributed interpolation points for baseline wander estimation and are therefore used on both synthetic and real data.

### 3.1 Synthetic data

Baseline wander can be modelled as a sinusoid around 0.15–0.3 Hz [8] that increases with exercise. Therefore, synthetic datasets are generated with 1 mV_p-p_ sinusoids each with fundamental frequencies that change from 0.05 to 0.7 Hz corresponding to a respiration rate of 3–42 per minute. These sinusoids are sampled at 360 Hz and last for 30 min (i.e. $ \simeq $650 k samples).

### 3.2 Real data

Real data is obtained from the MIT-BIH Noise Stress Database [9]. These datasets (*BWM1.mat* and *BWM2.mat*) are baseline wander recordings, sampled at 360 Hz with a gain of 200 V/V. Each recording lasts for 30 min (i.e. $ \simeq $650 k samples) and the fast fourier transform (FFT) of these signals show that the respiration frequency is mostly around 0.1 Hz with white Gaussian noise present throughout the whole sample set. Owing to this white noise, the baseline wander signals have been filtered with a 16-point moving average filter prior to testing. This filter order was deemed sufficient to reduce the white noise below 5 μV in worst conditions. Otherwise, the noise floor is defined by the white noise within the recorded signal itself making it impossible to test interpolation methods thoroughly.

## 4 Results and discussion

### 4.1 Synthetic data

**Fig. 2:**
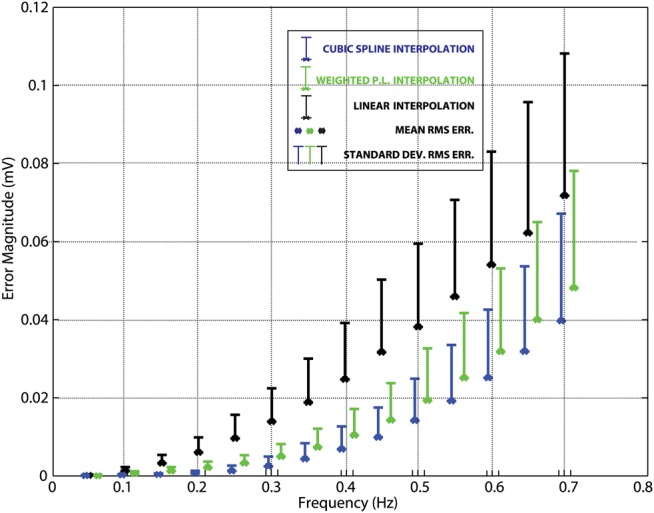
Synthetic data; mean and standard deviation of RMS errors per heartbeat of different interpolation methods

Fig. 2 compares three different interpolation methods: linear, cubic spline and our proposed interpolation method, when applied to sinusoidal baseline wander signals. Among all frequencies, WPL interpolation yields better accuracy when compared with linear interpolation alone. However, as the frequency of the sinusoids increase, all three algorithms’ performance degrades. This is due to there being less interpolation points per period available. When the respiration rate increases, this reflects an increase in pulse rate, maintaining a ratio of ∼1 breath for every 3–4 heartbeats [10] and on successful detection of fiducial points as in our previous work [7] at least 9–12 interpolation points should be located per period of the baseline wander. Using the 100 m.mat signal however, the heart rate of the patient is around 72 bpm, and as the frequency of the synthetic data increases less interpolation points can be used per period; therefore, we see a degradation in accuracy.

**Fig. 3:**
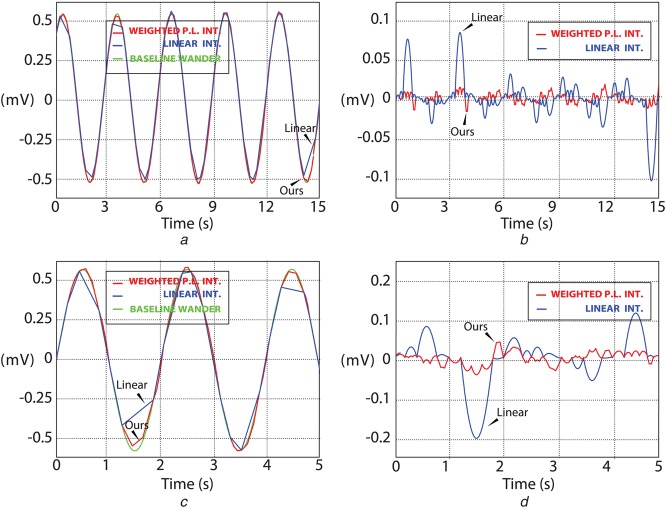
Comparison of our algorithm with linear interpolation using a 1 mV_p−p_ sinusoidal signal. Signals are denoted as sinusoidal (green), linear interpolation (blue), WPL interpolation (red). Shown are *a* 0.3 Hz sinusoid response *b* Sample-by-sample error analysis (linear versus WPL interpolation) *c* 0.5 Hz sinusoid response *d* Sample-by-sample error analysis (linear versus WPL interpolation)

Fig. 3 shows two sinusoids at different frequencies with their sample-by-sample errors for both WPL and linear interpolation. This shows that WPL interpolation successfully estimates curvatures when compared with linear. Even though, the algorithm does not perform better compared with cubic spline interpolation, the complexity required is significantly reduced without requiring second derivative calculations and triangular matrix solving for determining the polynomial coefficients.

### 4.2 Real data (MIT-BIH)

As described, the algorithm is also tested on baseline wander signals acquired from the MIT-BIH Noise Stress Database. Table 1 shows that BWM1 and BWM2 results differ. When we observed these two signals, BWM1 signal has a higher standard deviation (93 versus 36) and a higher kurtosis (15.6 versus 4.3) indicating BWM1 signal varies more in amplitude and peakedness which would make it more difficult to interpolate. Possible causes of this variance can be due to gender, stress test conditions and lung capacity since baseline wander occurs due to the impedance change seen by the amplifier as mentioned in the works of Friesen and *et.al.* [8]. Also, when comparing interpolation methods, we focused on both root mean square (RMS) and maximum errors since the baseline wander can be modelled as a sinusoid, RMS error would carry good measure of its effect, whereas maximum error seen during ST segment carries crucial information.

**Table 1:** Real data – RMS and maximum error per heartbeat and ST segment

Interpolation method	Signal, Hz	RMS error, μV per heartbeat^a^			Maximum Error, μV per ST segment^a^		
		*μ*	Median	*σ*	*μ*	Median	*σ*
Linear	BWM1	14.8	10.6	13.1	28.8	21.7	25.1
	BWM2	8.4	7.1	5.5	16.2	14.5	9.7
Cubic spline (windowed *N* = 3)	BWM1	13.5	9.2	14.2	26.1	19.3	21.8
	BWM2	7.9	6.4	5.4	15.3	13.6	8.0
WPL	BWM1	13.7	10.0	12.7	26.8	19.8	22.1
	BWM2	8.1	6.9	5.2	15.5	13.7	8.6

^a^2243 Heartbeats detected via MIT-BIH Arrythmia Database (100 m.mat)

**Fig. 4:**
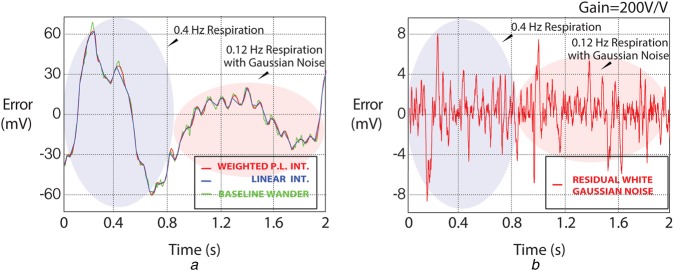
Error analysis between respiration rate versus residual Gaussian noise. Signals are denoted as real baseline wander (green), linear interpolation (blue), WPL interpolation (red). Shown are *a* BWM1.mat response *b* Sample-by-sample error analysis (WPL interpolation)

As mentioned in Section 3.2, the fundamental frequency of these signals is mostly around 0.1 Hz. On occasions where the respiration rate increases, the errors become more comparable with the residual Gaussian noise errors present. Fig. 4 shows a 0.12 Hz respiration signal with residual Gaussian noise comparable with the error results at 0.4 Hz respiration rate. This is due to the fact that, since less interpolation points can be used, any high-frequency content cannot be captured due to the Nyquist sampling theorem. Therefore, not all of the errors reported in Table 1 are due to interpolation errors.

**Fig. 5:**
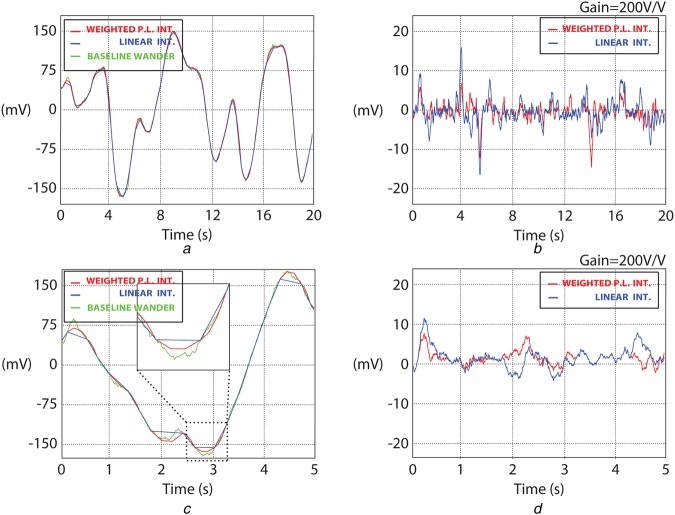
Comparison of our algorithm with linear interpolation with real baseline wander signals (BWM1.mat, BWM2.mat). Signals are denoted as real baseline wander (green), linear interpolation (blue), WPL interpolation (red). Shown are *a* BWM1.mat response *b* Sample-by-sample error analysis (linear versus WPL interpolation) *c* BWM2.mat response *d* Sample-by-sample error analysis (linear versus WPL interpolation)

**Fig. 6:**
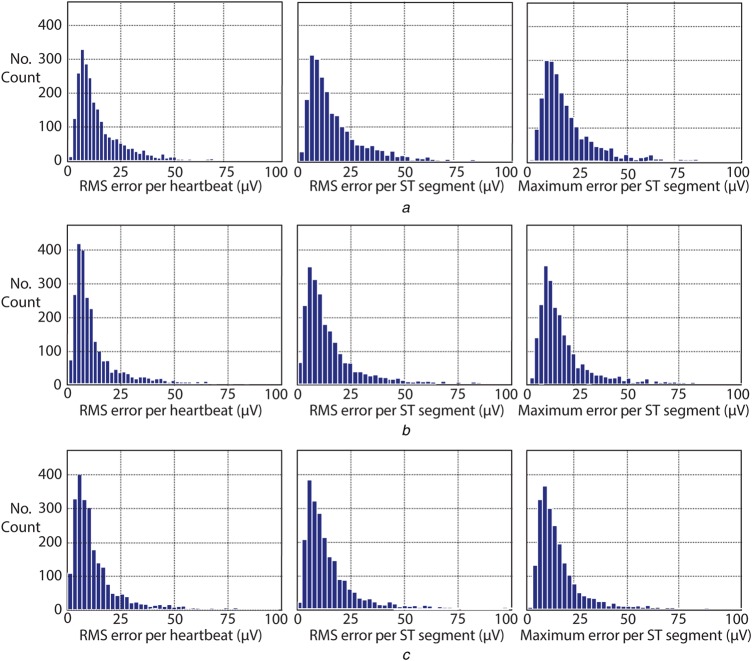
RMS and maximum error per heartbeat/ST segment histogram results of real baseline wander signal (BWM1.mat). Shown are for *a* Linear interpolation *b* Cubic spline interpolation *c* WPL interpolation

Fig. 5 shows that for almost all curves the algorithm results in smaller errors than linear interpolation except for one case, where an overshoot occurs, whereas Fig. 6 shows that the histogram of errors is more spread for linear interpolation, while WPL interpolation's spread more closely resembles that of cubic spline interpolation. In addition, these figures comply with American Heart Association and International Electrotechnical Commission standards which allow a maximum error of 100 μV for clinical ECG systems [11]. Also, Fig. 6 histogram results and Table 1 results show that RMS errors per heartbeat are in accordance with maximum ST segment errors. Even though all of these methods comply with the standards, in the event of missing fiducial point detections these errors would increase. This is also similar to Fig. 2 result; as the fiducial point count remained constant, frequency increase of the baseline wander degraded system performance due to decreased sampling rate.

### 4.3 Complexity

As the linear interpolation takes only two coordinates, the complexity of the WPL interpolation and cubic spline increases due to past knowledge requirements. In the case of WPL interpolation, the complexity of the algorithm is an additional slope calculation, interval segmentation and piecewise slope generation. As fiducial points are non-uniformly sampled depending on heart rate and characteristics (P, QRS and T waves) as mentioned in [7], an accurate complexity measure is hard to achieve. However, under normal conditions an estimation of interpolation point generation per 100 sample is a reasonable estimate. Table 2 shows the complexity requirements for each interpolation method under this assumption. As can be seen, WPL interpolation requires eight if statements, eight additions, two shift operations and four multiplications to generate a piecewise interpolation. The actual computational requirement on the other hand is much lower since the algorithm utilises these resources only when a turning point is detected. When we quantify the complexity measure of cubic spline interpolation, a single sample generation requires 14 floating point multiplications, 10 additions and 3 conditions [12] and also requires the solution of an *N* × *N* matrix to evaluate the second derivatives, where *N* is the window size defined for cubic spline interpolation. Therefore, the polynomial approach needs much more complexity; however, the advantages in return such as the continuity of the interpolation estimation get disturbed by the quantisation noise and the accuracy results do not show an effective improvement.

**Table 2:** Comparison in computational complexity between different interpolation methods

Interpolation method		Number of operations			
		Additions	Multiplications	Conditions	Memory
Linear	per sample	1	–	–	2
	per fiducial^a^	2	1	–	4
Cubic spline (windowed *N* = 3)	per sample	10	14	3	16
	per fiducial^a^	10 + 12*N*	10 + 10*N*	12 + 20*N*	4
WPL	per sample	2	–	4	2
	per fiducial^a^	6	6	4	12

^a^Fiducial points are detected every 100 samples under normal conditions.

## 5 Conclusion

In this Letter, we have described a computationally efficient interpolation algorithm that is suitable for real-time ECG baseline wander estimation. Using both synthetic and real data (from the MIT Noise Stress Database), we have shown that WPL interpolation is more accurate than linear interpolation, more computationally efficient than cubic spline interpolation and in compliance with clinically valid diagnosis.
